# Inflammatory storm and metabolic disorders: unraveling heterogeneity in mortality risk for comorbid diabetes mellitus and heart failure via the C-reactive protein-triglyceride-glucose index

**DOI:** 10.3389/fendo.2025.1689238

**Published:** 2025-11-19

**Authors:** Na Zhang, Lin Xie, Shuhua Zhang, Qun Wang, Hengcheng Lu, Zhiyu Xiong, Guobo Xie, Guotai Sheng, Hongyi Yang, Shiming He, Tanghong Liao, Wei Wang, Yang Zou

**Affiliations:** 1 Endocrinology Department, Jiangxi Provincial People’s Hospital, The First Affiliated Hospital of Nanchang Medical College, Nanchang, Jiangxi, China; 2 Jiangxi Cardiovascular Research Institute, Jiangxi Provincial People’s Hospital, The First Affiliated Hospital of Nanchang Medical College, Nanchang, Jiangxi, China; 3 Department of Cardiology, Jiangxi Provincial People’s Hospital, The First Affiliated Hospital of Nanchang Medical College, Nanchang, Jiangxi, China; 4 Discipline Construction Office, Jiangxi Provincial People’s Hospital, The First Affiliated Hospital of Nanchang Medical College, Nanchang, Jiangxi, China; 5 Jiangxi Medical College, Nanchang University, Nanchang, Jiangxi, China; 6 China-Australia Joint Research Center for Infectious Diseases, School of Public Health, Xi’an Jiaotong University Health Science Center, Xi’an, Shanxi, China

**Keywords:** C-reactive protein-triglyceride-glucose index, diabetes mellitus, acute decompensated heart failure, TyG index, C-reactive protein

## Abstract

**Introduction:**

Acute decompensated heart failure (ADHF), a critical cardiovascular emergency, is driven by a metabolic and inflammatory imbalance that serves as the central mechanism of disease progression. This study aims to analyze the heterogeneity of mortality risk in patients with comorbid diabetes mellitus (DM) and HF using the C-reactive protein-triglyceride-glucose index (CTI).

**Methods:**

This study evaluated 1,051 ADHF patients from the Jiangxi-ADHF II cohort. The Boruta algorithm, a fully automated feature selection method, was applied to identify key predictive variables and rank their importance. Cox proportional hazard models were constructed to assess the association between the CTI and 30-day mortality risk in ADHF patients, stratified by DM status. To further elucidate the nonlinear characteristics of risk associations, restricted cubic splines were employed to construct dose-response relationship curves. Additionally, heatmaps were used to assess the joint association of CTI components with mortality risk.

**Results:**

The 30-day follow-up revealed a mortality rate of 8.3%. Through the Boruta algorithm and multivariate Cox regression analysis, we identified CTI as a key prognostic factor for short-term mortality risk in ADHF patients, especially in those with comorbid DM. The restricted cubic splines model further confirmed the linear and non-linear associations between CTI and mortality in ADHF patients with and without DM. Additionally, heatmaps visualized the association between CTI components and mortality: to summarize, the mortality risk is relatively low when the triglyceride-glucose index remains within specific ranges (8.25-9.0 for patients with DM; 7.0-9.0 for non-DM patients) and the C-reactive protein level is maintained below 50 mg/L. Further subgroup analyses highlighted distinct risk modulation patterns: non-DM ADHF patients exhibited mortality risk heterogeneity across gender, hypertension, and stroke subgroups; however, the DM comorbid group demonstrated uniform risk profiles with no statistically significant differences.

**Discussion:**

This study demonstrates the clinical utility of the novel inflammatory-metabolic index CTI in mortality risk assessment for ADHF patients, with superior risk stratification efficacy observed in those with DM comorbidity.

## Graphical Abstract

Graphic Abstract

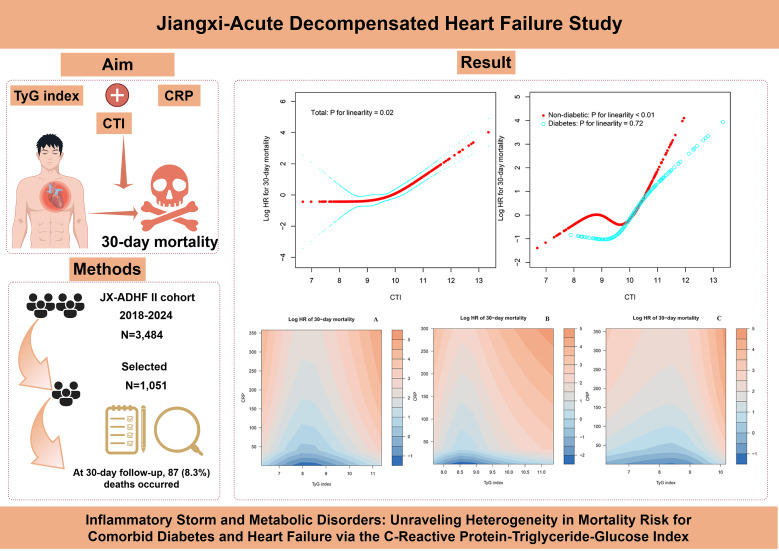

## Introduction

Diabetes mellitus (DM) stands as a major global public health challenge in the 21st century, imposing a growing disease burden. According to the 2019 Epidemiology Report by the International Diabetes Federation, the global prevalence of DM among adults reached 9.3% and is projected to rise to 10.9% by 2045 (1). Notably, cardiovascular complications contribute to the majority of (i.e., over 50%) mortality risk among people with DM (2). The Framingham study indicates that the risk of developing heart failure (HF) is significantly higher in DM patients than in non-DM individuals (3). Epidemiological evidence reveals the prevalence of HF among DM patients ranges from 9% to 22%, approximately fourfold higher than in the general population (4); conversely, the prevalence of DM among HF patients is between 10% and 47% (5). In terms of clinical prognosis, this comorbidity profile of metabolic and cardiovascular systems exhibits a significant superimposition of risks for adverse outcomes. Multivariable risk prediction models explicitly categorize DM as an independent risk factor for mortality in HF patients (6). Population-based longitudinal studies demonstrate that concomitant DM significantly elevates future mortality risks in HF populations (7–11). These evidence chains not only uncover the underlying pathological interconnections between DM-HF comorbidity but also underscore the clinical imperative of implementing dual intervention strategies in clinical management.

Acute decompensated HF (ADHF) represents a critical manifestation of cardiovascular disease. In recent years, fundamental research has elucidated that insulin resistance (IR) and chronic inflammation are the core pathological bases driving ADHF pathogenesis and progression. Specifically, IR, as a key driver of metabolic syndrome, not only directly impairs energy metabolism but also exacerbates myocardial fibrosis and diastolic dysfunction through pro-inflammatory cytokine release (12–14). Notably, chronic inflammation aggravates IR states via modulating autocrine effects in inflammatory cells and accelerating ectopic fat deposition, thereby creating a vicious cycle (15). Within this pathological framework, the discovery of novel biomarkers has provided transformative tools for ADHF risk stratification and prognostic assessment. The triglyceride-glucose (TyG) index, as a non-invasive quantitative tool for IR, has been incorporated into HF risk stratification systems due to its efficient IR characterization capability and clinical practicality (16). It has been validated as an independent predictor of mortality risks across various HF subtypes (17–23). Concurrently, C-reactive protein (CRP), recognized as a core mediator of systemic inflammation (24), has expanded its clinical significance beyond a marker for infectious diseases (25–35). Notably, recent studies have demonstrated that a combined assessment of metabolism and inflammation significantly enhances the predictive efficacy of cardiovascular event risk and adverse prognosis (36–39). This multidimensional assessment strategy not only transcends the limitations of traditional risk stratification models but also provides an actionable foundation for individualized interventions in ADHF management within the era of precision medicine.

The combined effect of metabolism and inflammation is a key driver in the progression of multisystem diseases (12–15). Building upon this, Ruan et al. developed the C-reactive protein-triglyceride-glucose index (CTI), which integrates CRP (an inflammatory marker) and the TyG index (a surrogate for IR), and demonstrated its independent predictive utility for mortality risk assessment (40). Subsequent investigations further revealed significant associations between CTI and adverse cardiocerebrovascular outcomes (41–43). However, in high-risk populations for acute cardiovascular events, particularly in DM-ADHF comorbid patients whose pathophysiological characteristics are closely linked to the metabolic-inflammatory axis, the predictive efficacy and threshold effects of CTI for short-term (30-day) mortality risk remain incompletely elucidated. To address this knowledge gap, this study, based on the Jiangxi-ADHF II cohort, aims to investigate the risk stratification capability of CTI for 30-day all-cause mortality in ADHF patients with and without comorbid DM, thereby providing evidence-based guidance for precision risk management of ADHF.

## Methods

### Study design and population

**Figure 1:**
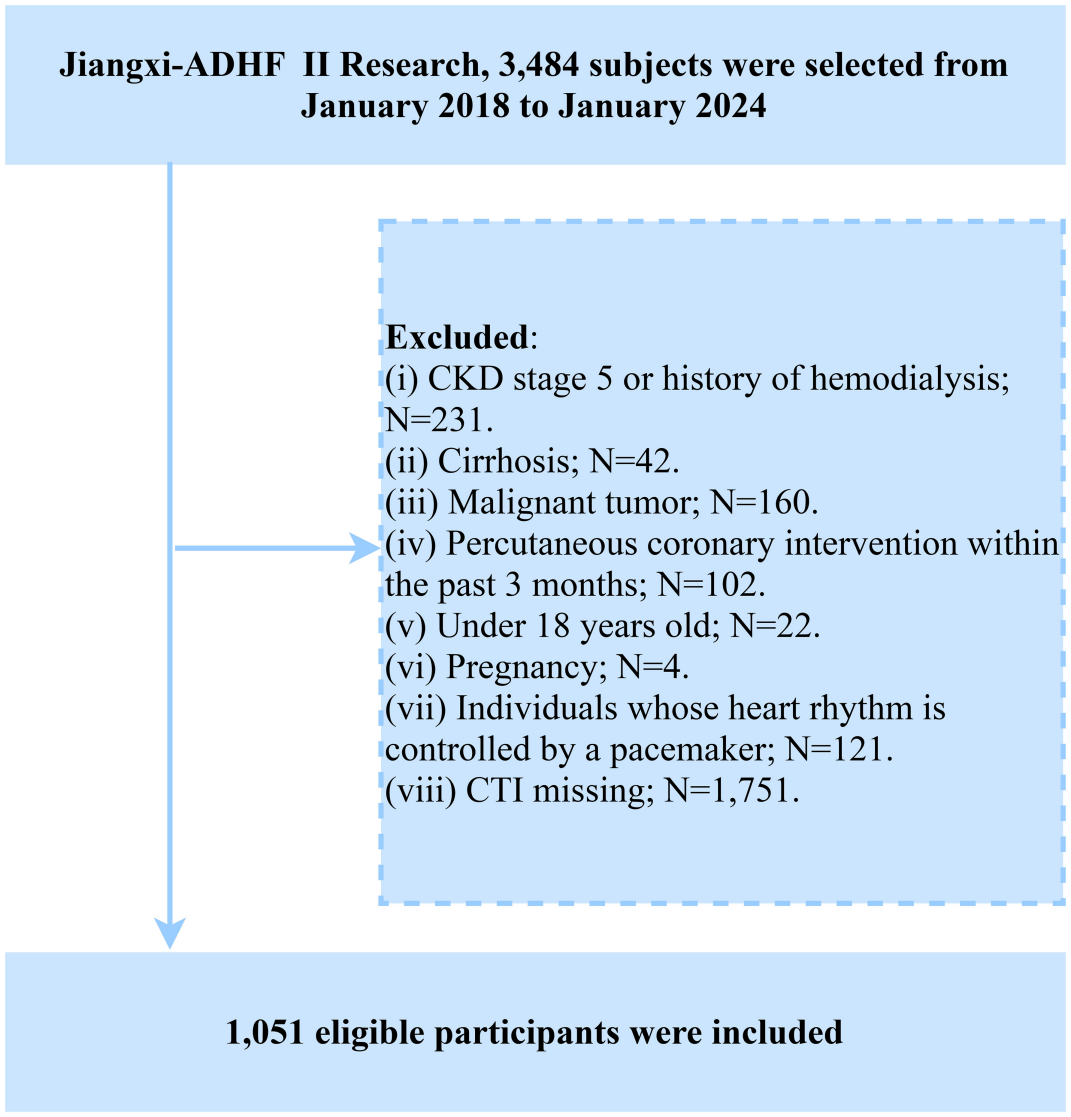
Flow chart for inclusion and exclusion of study participants.

The subjects of this study were recruited from the Jiangxi-ADHF cohort study. Briefly, the Jiangxi-ADHF project is an ongoing cohort study designed to integrate multidimensional clinical data for developing regional, standardized risk stratification models for HF. The Jiangxi-ADHF II cohort was conducted from January 2018 to January 2024, encompassing 3,484 patients with ADHF who met the diagnostic criteria outlined in the then-available guidelines of the European Society of Cardiology and the American College of Cardiology/American Heart Association for HF. We applied the following exclusion criteria to account for pathological heterogeneity and data integrity. Based on considerations of pathological heterogeneity and data integrity, subjects with the following characteristics were excluded: (1) Exclusion of individuals with fluid and sodium retention attributed to non-cardiac causes, including patients with uremia, chronic kidney disease requiring hemodialysis, and liver cirrhosis (n=273). (2) Exclusion of special treatment populations that may interfere with autonomic nervous regulation and short-term prognosis (including patients with pacemaker implantation and those who underwent interventional or reperfusion therapies within 30 days; n=223). (3) Exclusion of individuals with specific physiological states, including malignancies, minors, and pregnancy (n=186). (4) Exclusion of cases with missing CTI data (n=1,751). This ultimately included 1,051 participants in the final analysis, with the detailed screening process illustrated in **Figure 1**.

### Ethical approval

This study adhered strictly to international biomedical research ethical frameworks and received systematic evaluation and approval from the Ethics Review Committee of Jiangxi Provincial People’s Hospital prior to implementation (Ethics Approval No: 2024-01). In accordance with the ethical principles outlined in the Declaration of Helsinki, informed consent was obtained from all participants or their legal guardians for data utilization. The study’s findings are reported in compliance with the Strengthening the Reporting of Observational Studies in Epidemiology guidelines.

### Data collection

In the clinical data acquisition phase, this study employed a dual independent data entry-blinded verification quality control system: two standardized-trained research assistants independently collected demographic characteristics (gender, age), lifestyle factors (smoking and drinking status), cardiovascular comorbidities [hypertension, DM, stroke, coronary heart disease (CHD)], New York Heart Association (NYHA) functional classification, vital signs (blood pressure), and echocardiographic parameters (left ventricular ejection fraction: LVEF). Following cross-verification between the two datasets, validated information was included in the final analysis. Concurrently, comorbidity diagnoses were substantiated through a comprehensive review of patients' electronic medical records containing specialist consultation records, medication histories, and supporting imaging evidence.

The laboratory analysis incorporated blood sample data collected within 24 hours of admission, with strict fasting criteria implemented for glucose/lipid metabolism-related indicators and liver enzyme tests (minimum interval of ≥8 hours from last meal to blood sampling). Metabolic parameters were measured using the HITACHI LABOSPECT 008 fully automated biochemical analyzer, including liver/kidney function markers [alanine aminotransferase (ALT), aspartate aminotransferase (AST), creatinine (Cr), uric acid (UA)], and glucose/lipid profiles [fasting plasma glucose (FPG), total cholesterol, triglycerides (TG), low-density lipoprotein cholesterol (LDL-C), high-density lipoprotein cholesterol (HDL-C)]. Blood cell (WBC: white blood cell count; RBC: red blood cell count; PLT: platelet count) analysis was performed using the Sysmex XN-3000 5-part differential hematology analyzer. The HF biomarker N-Terminal Pro-Brain Natriuretic Peptide (NT-proBNP) was quantitatively measured via electrochemiluminescence immunoassay. CRP levels were quantified using an immunoturbidimetric assay. All testing protocols were monitored through standardized quality control protocols to ensure result reliability.

### Calculation of TyG index and CTI

TyG index = ln [TG (mg/dL) × FPG (mg/dL)/2] (16).

CTI = CTI = 0.412 × Ln (CRP [mg/L]) + TyG index (40).

### Study outcomes

This study used the time of admission for ADHF patients as the observation starting point, with the primary outcome defined as all-cause mortality occurring within 30 days. Outcome ascertainment was conducted through multiple methods, including follow-up via mobile text messages, telephone interviews, and in-person follow-up during outpatient or inpatient visits, all performed by medically trained personnel. In addition to in-hospital deaths, out-of-hospital deaths have also been systematically verified.

### Missing data processing

In the current study, there are missing data for LVEF, ALT, AST, Cr, and UA, with missing rates of 4.00%, 0.57%, 0.57%, 0.95%, and 1.05% respectively. The detailed information is provided in **Supplementary Table 1**. Analysis of the missingness pattern cross-information diagram (**Supplementary Figure 1**) revealed high correlations in missing patterns between UA and Cr, as well as ALT and AST, suggesting these data are likely missing at random. Conversely, LVEF demonstrated relative independence from other missing variables, indicating its missing data might be missing completely at random. Given the low missing proportion and the observed independence, we used the mice package in R software to perform multiple imputation for missing covariate information: predictive mean matching was used for continuous variables, while logistic regression was applied to categorical variables.

### Statistical analysis

This study utilized Free Statistics 1.7, R language 3.4.1, and Empower(R) 2.0 software platforms for data analysis. Participants were stratified into tertile-based groups (low, moderate, high) according to CTI distribution. Baseline characteristics were described following variable-specific conventions: categorical variables as frequencies (percentages), normally distributed continuous variables as mean ± standard deviation, and non-normally distributed continuous variables as median (interquartile range). Between-group comparisons were conducted using chi-square tests, one-way Analysis of Variance, Student's t-test, or non-parametric tests as appropriate. A two-sided significance threshold of p < 0.05 was uniformly applied for all statistical inferences.

In the feature selection phase, the Boruta algorithm was employed to identify potential biomarkers associated with all-cause mortality in ADHF from high-dimensional clinical data (44). In detail, the Boruta method is based on a random forest model and identifies statistically significant features by comparing the importance of "real features" with that of "shadow features". The core steps are as follows: Shadow features with the same distribution as real features are generated (original feature values are randomly shuffled to eliminate their real association with the outcome). Both real and shadow features are input into the random forest model together, and feature importance is evaluated through repeated iterations ("Gini impurity decrease" was used as the importance metric in this study); if the importance of a real feature is significantly higher than that of all shadow features (assessed by Z-test), it is classified as an "important feature"; otherwise, it is labeled as "unimportant" or "tentative".

To optimize model stability, a dual-stage variable screening strategy was implemented: (1) variables exhibiting multicollinearity were first excluded using the variance inflation factor method (**Supplementary Table 2**), followed by (2) elimination of CTI components (e.g., TG, FPG) considering underlying collinearity concerns. Survival analysis was conducted using the Kaplan-Meier method, generating survival curves and performing log-rank tests, with the proportional hazards assumption visually validated. Three progressively adjusted Cox proportional hazards models were subsequently constructed: Model I was adjusted for demographic parameters (gender, age, smoking, and drinking status); Model II was further adjusted for clinical comorbidities (hypertension, stroke, CHD), NYHA functional class, and LVEF; As the final model, Model III was built upon Model II by additionally adjusting for hematological and functional laboratory parameters, including RBC, PLT, AST, Cr, UA, LDL-C, and NT-proBNP. All analyses were stratified by DM comorbidity status.

To elucidate the nonlinear relationship between CTI and prognosis, this study innovatively integrated multiple visualization techniques. We used restricted cubic spline (RCS) curves with 4 knots at the 5th, 35th, 65th, and 95th percentiles to visualize the dose-response relationship between CTI and mortality. The CTI inflection points where the association changed were identified using a recursive algorithm and piecewise Cox regression. For the knot selection in the RCS analysis, we followed the recommendations of Professor Harrell in Regression Modeling Strategies (45): Four knots provide an optimal balance, ensuring both curve smoothness and protection against overfitting, thereby preventing a loss of precision. Five knots may be more suitable for larger sample sizes, while 3 knots are recommended for small samples (n < 30). We also utilized a heatmap to reveal the joint influence of CTI components on mortality risk. This approach intuitively displays the interplay between inflammatory and metabolic factors in mortality risk evaluation (46).

Further subgroup analyses evaluated potential effect modifiers, including age, gender, cardiac function (LVEF/NYHA classification), and comorbidities (hypertension, stroke, and CHD). First, a stratified analysis was performed to assess the association between CTI and mortality in ADHF patients across different subpopulations. Then, the likelihood ratio test was used to quantify the differences between groups and determine the presence of interaction effects, with particular attention to the differential results in patients with and without DM.

To clarify the clinical application value of CTI, we further compared it with the existing HF risk score, ADHERE (Acute Decompensated Heart Failure National Registry) (47), to evaluate its predictive performance for short-term mortality outcomes. The evaluation metrics included the area under the curve and the net reclassification improvement to determine its incremental predictive value.

### Sensitivity analysis

To test the robustness of the association between CTI and mortality, we conducted several sensitivity analyses: (1) For external validation, we used data from the U.S. (United States) National Health and Nutrition Examination Survey between 1998 and 2018 to analyze the association between CTI and all-cause mortality in U.S. patients with congestive HF. (2) To control for potential confounding by relevant medications, we adjusted for the use of statins, sodium-glucose cotransporter-2 inhibitors, and anti-inflammatory drugs in the multivariable model. (3) We further distinguished the types of study outcomes and evaluated the association between CTI and cardiovascular-specific mortality events.

## Results

### Baseline characteristics of the study population are presented according to CTI groups

This study enrolled 1,051 eligible ADHF patients, including 611 males (58.14%) and 440 females (41.86%), with a mean age of 69 years. Clinical characteristics of the study population were stratified by CTI tertiles (**Table 1**). Demographic analysis revealed a distinct gender distribution pattern in the high CTI group (9.87-13.34), with a significantly higher proportion of males. Comorbidity analysis showed that patients in the high CTI group not only had significantly elevated rates of hypertension, DM, and CHD but also demonstrated more severe hemodynamic profiles. Baseline characteristics of the high CTI group were further corroborated by multi-parameter laboratory assessments. While inflammatory markers showed significant elevations in WBC count and PLT count, metabolic profiles revealed more pronounced glucolipid metabolic dysregulation, along with hepatorenal dysfunction, characterized by significantly higher levels of FPG, TG, total cholesterol, LDL-C, ALT, Cr, UA, NT-proBNP, and lower HDL-C. Notably, patients with ADHF exhibited a progressive upward trend in TyG index and CRP levels, with significant between-group differences observed.

**Table 1:** Summary of baseline characteristics of the study population according to the CTI tertiles group.

Variable	CTI tertiles group			*P*-value
	Low (6.69-9.11)	Moderate (9.12-9.87)	High (9.87-13.34)	
No. of subjects	350	350	351	
Age (years)	71.00 (60.00-80.00)	71.00 (60.00-80.00)	72.00 (59.00-80.00)	0.88
Gender (n, %)				0.01
Male	187 (53.43)	199 (56.86)	225 (64.10)	
Female	163 (46.57)	151 (43.14)	126 (35.90)	
Comorbidities				
Hypertension (n, %)	128 (36.57)	163 (46.57)	176 (50.14)	<0.01
Diabetes (n, %)	46 (13.14)	90 (25.71)	145 (41.31)	<0.01
Stroke (n, %)	61 (17.43)	58 (16.57)	78 (22.22)	0.12
CHD (n, %)	84 (24.00)	108 (30.86)	132 (37.61)	<0.01
NYHA classification (n, %)				<0.01
III	254 (72.57)	226 (64.57)	210 (59.83)	
IV	96 (27.43)	124 (35.43)	141 (40.17)	
Drinking status (n, %)				0.28
No	312 (89.14)	317 (90.57)	325 (92.59)	
Yes	38 (10.86)	33 (9.43)	26 (7.41)	
Smoking status (n, %)				0.49
No	297 (84.86)	292 (83.43)	286 (81.48)	
Yes	53 (15.14)	58 (16.57)	65 (18.52)	
LVEF (%)	49.00 (39.00-56.75)	48.00 (38.00-56.00)	48.00 (39.00-56.50)	0.53
WBC (×10^9^/L)	5.50 (4.39-6.72)	6.57 (5.20-8.32)	7.60 (5.75-10.72)	<0.01
RBC (×10^12^/L)	4.05 (3.59-4.49)	4.03 (3.57-4.56)	3.98 (3.46-4.45)	0.36
PLT (×10^9^/L)	146.00 (111.25-188.00)	169.00 (133.25-214.75)	182.00 (136.50-239.00)	<0.01
ALT (U/L)	20.00 (13.00-32.75)	22.50 (14.00-42.00)	23.00 (14.50-44.00)	<0.01
AST (U/L)	25.00 (20.00-37.00)	27.00 (20.00-40.75)	28.00 (19.00-47.00)	0.17
Cr (umol/L)	79.00 (64.00-102.00)	90.00 (73.00-125.50)	101.71 (75.00-157.00)	<0.01
UA (umol/L)	398.00 (313.50-480.00)	429.50 (339.00-545.00)	440.00 (323.00-590.50)	<0.01
TG (mmol/L)	0.88 (0.73-1.09)	1.18 (0.95-1.51)	1.52 (1.11-2.04)	<0.01
TC (mmol/L)	3.47 (2.97-4.21)	3.80 (3.28-4.49)	3.83 (3.13-4.56)	<0.01
HDL-C (mmol/L)	1.05 (0.87-1.24)	0.99 (0.79-1.19)	0.87 (0.71-1.07)	<0.01
LDL-C (mmol/L)	2.04 (1.63-2.58)	2.25 (1.83-2.89)	2.30 (1.80-2.95)	<0.01
FPG (mmol/L)	5.06 (1.12)	5.58 (1.35)	7.28 (3.63)	<0.01
NT-proBNP (pmol/L)	3079.00 (1612.00-5205.75)	3742.50 (1858.50-6755.00)	3977.00 (1625.50-7358.50)	<0.01
TyG index	8.17 (0.38)	8.56 (0.42)	9.03 (0.60)	<0.01
CRP (mg/L)	2.99 (1.54-5.39)	9.50 (4.85-17.00)	41.00 (15.80-92.25)	<0.01
30-day mortality (n, %)	13 (3.71)	19 (5.43)	55 (15.67)	<0.01

CHD, coronary heart disease; NYHA, New York Heart Association; LVEF, left ventricular ejection fraction; WBC, white blood cell count; RBC, red blood cell count; PLT, platelet count; TG, triglyceride; TC, total cholesterol; HDL-C, high-density lipoprotein cholesterol; LDL-C, low-density lipid cholesterol; Cr, creatinine; ALT, alanine aminotransferase; AST, aspartate aminotransferase; NT-proBNP, N-Terminal Pro-Brain Natriuretic Peptide; UA, uric acid; FPG, fasting plasma glucose; CRP, C reactive protein; TyG, triglyceride-glucose; CTI, C-reactive protein-triglyceride-glucose index.

**Table 2** further stratifies the clinical baseline characteristics of ADHF patients with or without DM. Compared to non-DM patients, ADHF patients with comorbid DM exhibited a more complex comorbidity network, characterized by higher comorbidity rates of hypertension and CHD, along with more severe cardiac dysfunction deterioration. Laboratory profiles of patients with comorbid DM demonstrated a characteristic metabolic dysregulation pattern: in addition to significant elevations in WBC count, PLT count, FPG, and TG, higher levels of ALT and Cr suggested multi-organ involvement. Critically, both CRP and TyG index were significantly higher in DM patients.

**Table 2:** Summarize the baseline characteristics of the study population according to whether they are complicated with diabetes or not.

Variable	Non-diabetic group	Diabetes group	P-value
No. of subjects	770	281	
Age (years)	71.00 (59.00-80.00)	71.00 (60.00-80.00)	0.96
Gender (n, %)			0.61
Male	444 (57.66)	167 (59.43)	
Female	326 (42.34)	114 (40.57)	
Comorbidities			
Hypertension (n, %)	296 (38.44)	171 (60.85)	<0.01
Stroke (n, %)	138 (17.92)	59 (21.00)	0.26
CHD (n, %)	205 (26.62)	119 (42.35)	<0.01
NYHA classification (n, %)			<0.01
III	528 (68.57)	162 (57.65)	
IV	242 (31.43)	119 (42.35)	
Drinking status (n, %)			0.99
No	699 (90.78)	255 (90.75)	
Yes	71 (9.22)	26 (9.25)	
Smoking status (n, %)			0.70
No	639 (82.99)	236 (83.99)	
Yes	131 (17.01)	45 (16.01)	
LVEF (%)	49.00 (38.00-56.00)	48.00 (39.00-56.00)	0.46
WBC (×10^9^/L)	6.22 (4.83-7.90)	7.20 (5.60-9.50)	<0.01
RBC (×10^12^/L)	4.02 (3.60-4.49)	3.99 (3.47-4.50)	0.51
PLT (×10^9^/L)	157.50 (123.00-204.75)	179.00 (137.00-229.00)	<0.01
ALT (U/L)	22.00 (14.00-41.00)	22.00 (13.00-37.00)	0.53
AST (U/L)	27.00 (20.00-41.75)	24.00 (19.00-37.00)	<0.01
Cr (umol/L)	86.00 (68.00-119.00)	100.00 (75.00-153.00)	<0.01
UA (umol/L)	420.00 (329.00-536.75)	419.00 (325.00-544.00)	0.78
TG (mmol/L)	1.09 (0.83-1.47)	1.29 (1.00-1.77)	<0.01
TC (mmol/L)	3.68 (3.11-4.36)	3.79 (3.03-4.44)	0.42
HDL-C (mmol/L)	0.99 (0.78-1.19)	0.94 (0.75-1.11)	<0.01
LDL-C (mmol/L)	2.20 (1.76-2.79)	2.23 (1.72-2.80)	0.80
FPG (mmol/L)	5.25 (1.08)	7.97 (3.87)	<0.01
NT-proBNP (pmol/L)	3393.00 (1696.50-6339.25)	3890.00 (1718.00-6914.00)	0.28
TyG index	8.44 (0.50)	8.96 (0.64)	<0.01
CRP (mg/L)	7.75 (3.19-27.04)	10.70 (4.09-33.10)	0.01
CTI	9.37 (0.81)	9.98 (0.90)	<0.01
30-day mortality (n, %)	55 (7.14)	32 (11.39)	0.03

CHD, coronary heart disease; NYHA, New York Heart Association; LVEF, left ventricular ejection fraction; WBC, white blood cell count; RBC, red blood cell count; PLT, platelet count; TG, triglyceride; TC, total cholesterol; HDL-C, high-density lipoprotein cholesterol; LDL-, low-density lipid cholesterol; Cr, creatinine; ALT, alanine aminotransferase; AST, aspartate aminotransferase; NT-proBNP, N-Terminal Pro-Brain Natriuretic Peptide; UA, uric acid; FPG, fasting plasma glucose; CRP, C reactive protein; TyG, triglyceride-glucose; CTI, C-reactive protein-triglyceride-glucose index.

### Follow-up results

**Figure 2:**
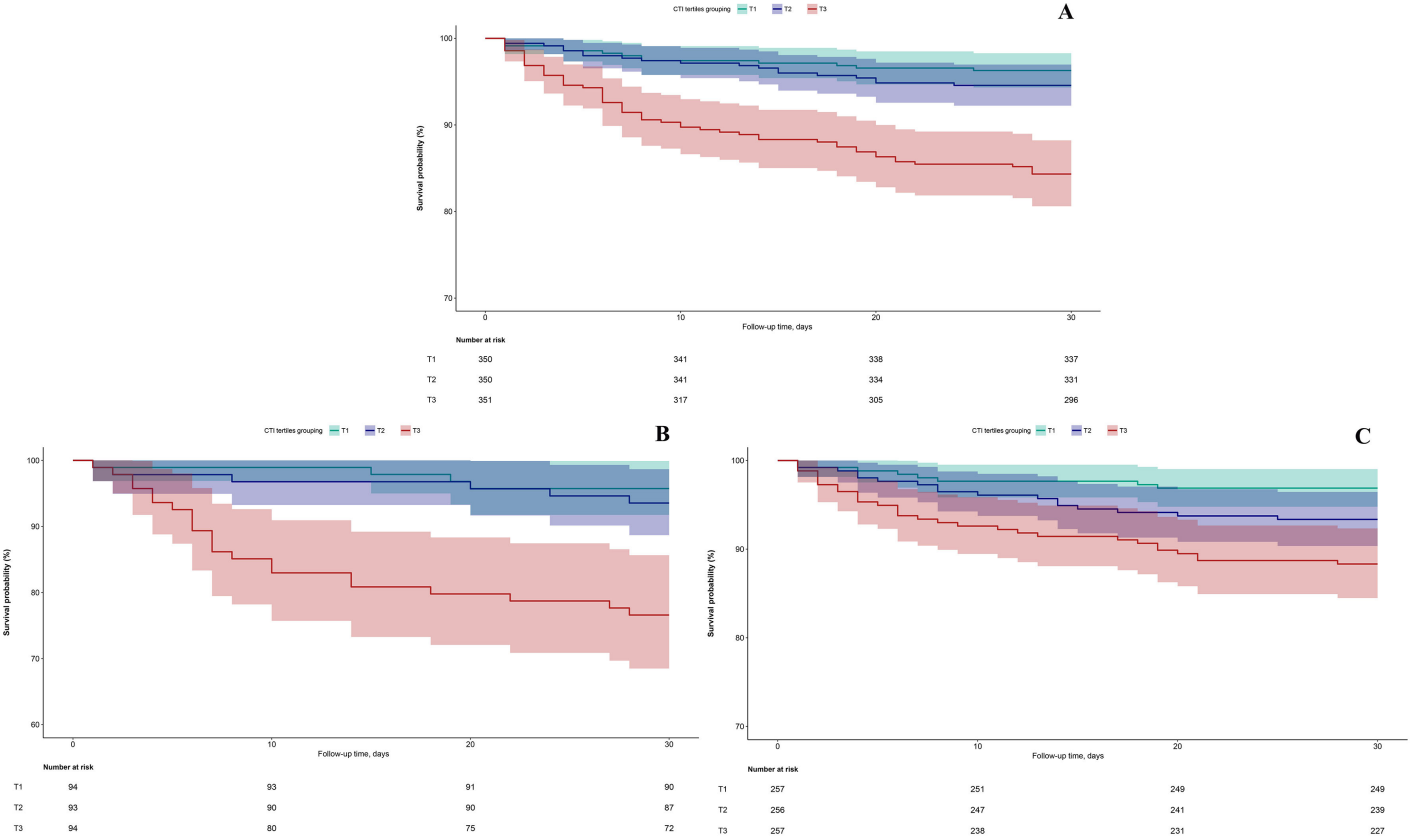
30-day survival curves of ADHF patients stratified by CTI tertiles **(A)** Total; **(B)** Diabetes group; **(C)** Non-diabetic group. ADHF: acute decompensated heart failure; CTI: C-reactive protein-triglyceride-glucose index.

A 30-day prognostic follow-up was conducted for 1,051 ADHF patients, during which 87 (8.3%) all-cause death events were observed. Survival analysis based on CTI tertile grouping demonstrated that ADHF patients in the high CTI group exhibited significantly higher all-cause mortality compared to those in the low and middle CTI groups (**Figure 2**, Log-rank *p* < 0.0001). Notably, this trend persisted in subgroup analyses of patients with and without DM, with DM patients in the high CTI group showing markedly lower survival rates than non-DM patients (**Figures 2B, C**).

### Feature selection

**Figure 3:**
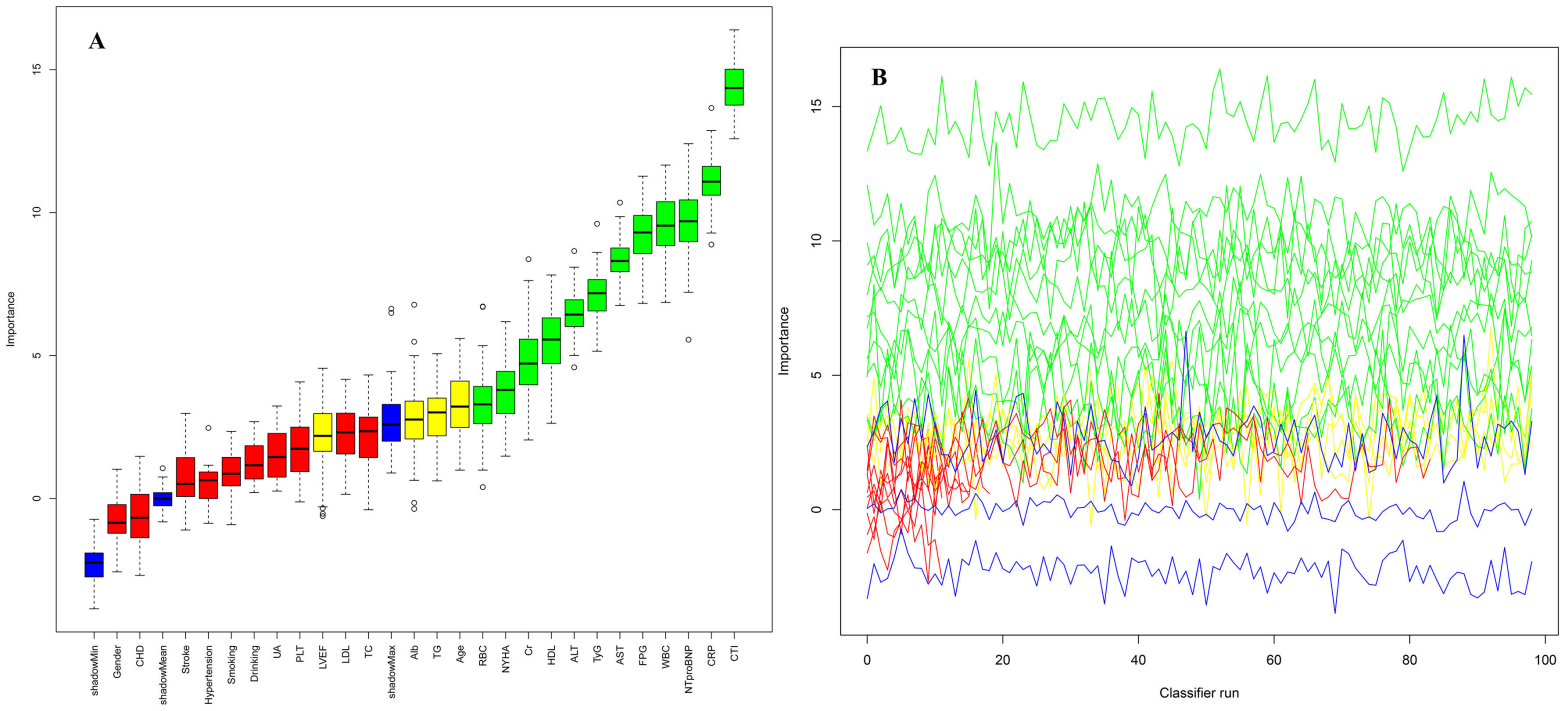
Feature selection for 30-day mortality in ADHF patients using the Boruta algorithm. **(A)** The process of feature selection; **(B)** The value evolution of the Z-score in the screening process. The horizontal axis shows the name of each variable and the number of times the classifier is run in **(A, B)** respectively. The vertical axis represents the Z-value of each variable. The green boxes and lines represent confirmed variables, the yellow ones represent tentative attributes, and the red ones represent rejected variables in the model calculation.

This study systematically identified predictors significantly associated with 30-day all-cause mortality in ADHF patients using the Boruta ensemble feature selection algorithm (**Figure 3**). By iteratively comparing the importance scores of original features against randomly generated "shadow features," the Boruta algorithm ultimately confirmed 12 critical predictors of mortality in ADHF patients, including RBC count, NYHA classification, Cr, HDL-C, ALT, TyG index, AST, FPG, WBC count, NT-proBNP, CRP, and the CTI index. Notably, among all significant features, the CTI index demonstrated the highest importance score (Z-score ≈ 15), significantly outperforming other variables and underscoring its dominant role in prognostic assessment for ADHF patients. To assess the stability of the feature selection results, we employed the bootstrap method to generate 100 bootstrap samples. The Boruta algorithm was run independently on each sample, and the frequency of each feature being selected across all runs was recorded. The results showed that the features included in our final report were consistently selected in over 90% of the runs, which fully demonstrates that they are not the result of random fluctuations but rather important features with a robust association with the outcome variable.

### Association between CTI and 30-day mortality in ADHF patients across overall, DM, and non-DM populations

Multivariable Cox proportional hazards model analyses (**Table 3**) revealed a significant positive association between CTI and 30-day all-cause mortality risk in ADHF patients. The prognostic assessment of CTI as a continuous variable demonstrated robustness across three adjusted models: in the base model (adjusted for demographic characteristics), each 1-unit increase in CTI was associated with a 193% elevated mortality risk [Hazard ratios (HR): 2.93: 2.31, 3.71]. Subsequent adjustment for comorbidities and cardiac function (NYHA classification and LVEF) attenuated the HR to 2.78 (2.19, 3.52). In the fully adjusted model (Model III) incorporating hematological parameters, CTI remained significantly and positively associated with mortality risk (HR: 2.68: 2.09, 3.44). CTI-stratified analysis further highlighted its risk gradient effect: compared to the low CTI group, patients in the high CTI group exhibited a 184% increased mortality risk (HR: 2.84: 1.48, 5.43).

**Table 3:** Multivariable Cox regression analysis of the associations between CTI and 30-day mortality in patients with ADHF.

Independent variable	No. of subjects (n)	No. of cases (n, %)	HR (95% CI)		
			Model I	Model II	Model III
Total					
CTI			2.93 (2.31, 3.71)	2.78 (2.19, 3.52)	2.68 (2.09, 3.44)
CTI tertiles					
T1(Low)	350	13 (3.71)	1.00	1.00	1.00
T2(Moderate)	350	19 (5.43)	1.44 (0.71, 2.92)	1.32 (0.65, 2.68)	1.01 (0.49, 2.10)
T3(High)	351	55 (15.67)	4.23 (2.27, 7.89)	3.59 (1.93, 6.70)	2.84 (1.48, 5.43)
Diabetes Group					
CTI			3.07 (2.19, 4.32)	3.26 (2.29, 4.63)	3.34 (2.24, 5.00)
CTI tertiles					
T1(Low)	46	1 (2.17)	1.00	1.00	1.00
T2(Moderate)	90	4 (4.44)	2.09 (0.23, 18.92)	2.38 (0.26, 21.71)	2.10 (0.22, 19.92)
T3(High)	145	27 (18.62)	9.40 (1.25, 70.58)	9.98 (1.32, 75.25)	8.71 (1.10, 68.74)
Non-diabetic group					
CTI			2.90 (2.06, 4.09)	2.79 (1.95, 4.01)	2.62 (1.76, 3.89)
CTI tertiles					
T1(Low)	304	12 (3.95)	1.00	1.00	1.00
T2(Moderate)	260	15 (5.77)	1.44 (0.68, 3.09)	1.18 (0.55, 2.53)	0.76 (0.33, 1.73)
T3(High)	206	28 (13.59)	3.52 (1.78, 6.98)	2.98 (1.49, 5.97)	2.25 (1.09, 4.63)

HR, hazard ratios; CI, confidence interval; ADHF, acute decompensated heart failure; CTI, C-reactive protein-triglyceride-glucose index.

Model I adjusted for gender, age, drinking status, and smoking status.

Model II adjusted for model I + hypertension, stroke, CHD, NYHA classification, and LVEF.

Model III adjusted for: Model II + RBC, PLT, AST, Cr, UA, LDL-C, and NT-proBNP.

Notably, the CTI-associated mortality risk was significantly higher in DM patients. Specifically, each 1-unit increase in CTI corresponded to an elevated mortality risk in patients with comorbid ADHF and DM (HR: 3.34: 2.24, 5.00) compared to non-DM ADHF patients (HR: 2.62: 1.76, 3.89). When stratified by CTI tertiles, this glucose metabolism-dependent risk disparity became more pronounced: ADHF patients with DM in the high CTI group exhibited an 8.71-fold increased mortality risk compared to the low CTI group; significantly exceeding the 2.25-fold risk observed in non-DM patients.

### Dose-response association between CTI and 30-day mortality in ADHF patients across overall, DM, and non-DM populations

**Figure 4:**
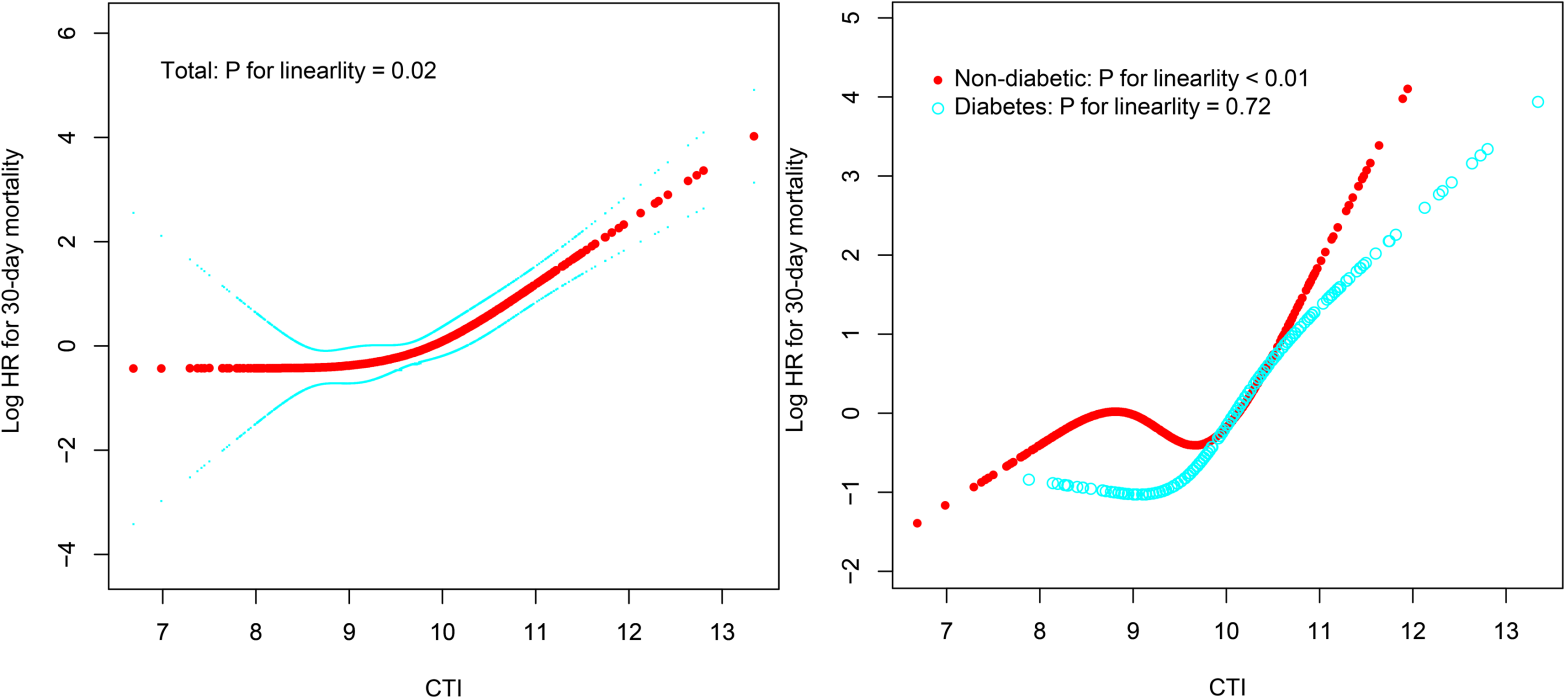
Fitting the dose-response relationship between CTI and 30-day all-cause mortality in ADHF patients with 4 knots restricted cubic spline. ADHF: acute decompensated heart failure; CTI: C-reactive protein-triglyceride-glucose index.

This study employed RCS analysis to further elucidate the dose-response association between CTI and 30-day all-cause mortality in ADHF patients (**Figure 4**). After adjusting for confounding factors, including demographic characteristics, comorbidities, cardiac function, and hematological parameters, a significant obtuse-angled nonlinear association between CTI and 30-day all-cause mortality was observed in the overall population (*P* for nonlinearity = 0.02). The inflection point value was calculated as 9.83 using recursive algorithms (**Table 4**), suggesting a potential threshold effect in the risk model. Specifically, when CTI < 9.83, ADHF patients exhibited a gradual increase in 30-day all-cause mortality risk (HR: 1.10: 0.64, 1.91). However, when CTI > 9.83, the risk curve steepened dramatically (HR: 3.37: 2.55, 4.46), with a 3.06-fold higher risk increment compared to the low-CTI segment (*P* for likelihood ratio test < 0.01).

**Table 4:** The result of the two-piecewise Cox regression model.

Independent variable	HR (95%CI)	*P*-value
Total		
The inflection point of CTI	9.83	
< 9.83	1.10 (0.64, 1.91)	0.73
> 9.83	3.37 (2.55, 4.46)	<0.01
*P* for likelihood test	<0.01	
Non-diabetic group		
The inflection point of CTI	10.04	
< 10.04	0.88 (0.50, 1.56)	0.66
> 10.04	10.31 (5.02, 21.18)	<0.01
*P* for likelihood test	<0.01	

HR, hazard ratios; CI, confidence interval; CTI, C-reactive protein-triglyceride-glucose index.

Adjusted for gender, age, drinking status, smoking status, hypertension, stroke, CHD, NYHA classification, LVEF, RBC, PLT, AST, Cr, UA, LDL-C, and NT-proBNP.

The study further evaluated the dose-response association between CTI and 30-day all-cause mortality risk in ADHF patients with and without DM. Results demonstrated a nonlinear association between CTI and all-cause mortality risk in non-DM ADHF patients (*P* for nonlinearity < 0.01), with an inflection point at 10.04 (**Table 4**). Notably, the high-CTI group exhibited a steeper risk escalation (HR: 10.31 vs. 0.88, *P* for likelihood ratio test < 0.01). In stark contrast, among ADHF patients with DM, CTI demonstrated a positive linear association with all-cause mortality risk (*P* for nonlinearity = 0.72).

### Exploratory analysis of the combined association between TyG and CRP (components of CTI) and 30-day mortality in ADHF patients across overall, DM, and non-DM populations

**Figure 5:**
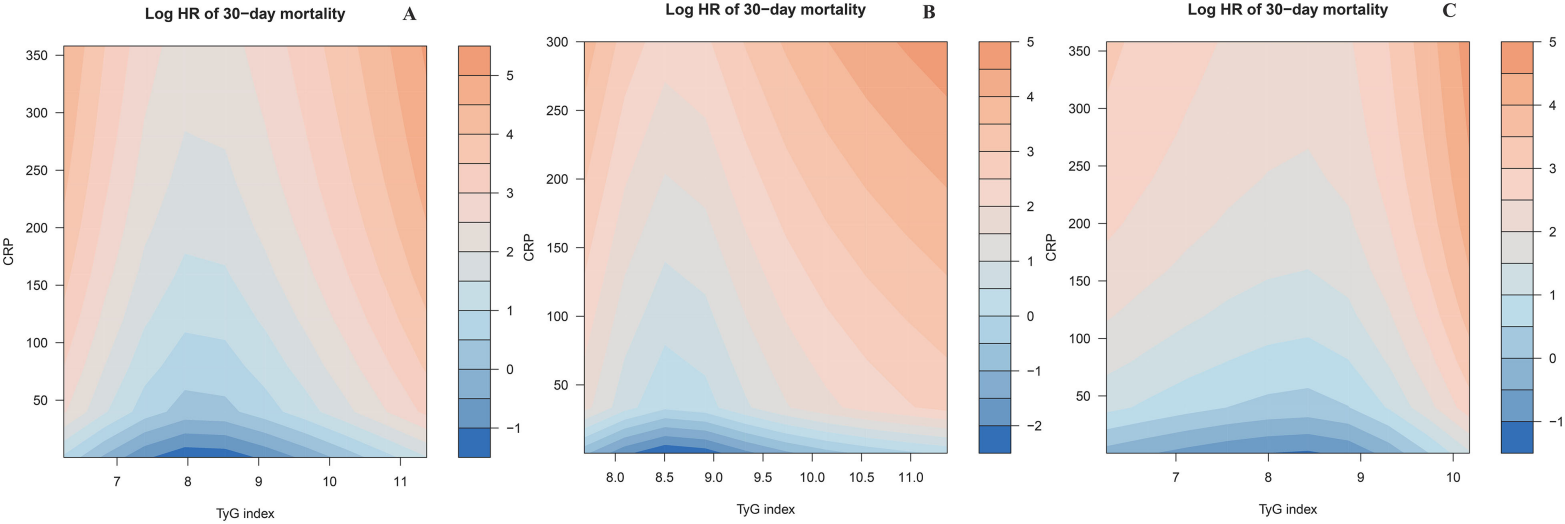
Heatmap of potential interaction effect between TyG index and CRP on 30-day all-cause mortality in ADHF patients **(A)** Total; **(B)** Diabetes group; **(C)** Non-diabetic group). The color gradient represents the combined effect of TyG index and CRP levels on mortality risk: the deeper the blue, the stronger the negative association with mortality, while the deeper the orange, the stronger the positive association with mortality. CRP: C-reactive protein; TyG index: triglyceride-glucose index; ADHF: acute decompensated heart failure.

Based on the adjustment strategy of Model III, we identified the potential interactive association between CTI components (TyG index and CRP) and 30-day mortality risk in ADHF patients using heatmaps. As shown in **Figure 5**, for the total population, the mortality risk is lowest (indicated by the darker blue band) when the TyG index remains between 7.5 and 9 and the CRP level is maintained below 50 mg/L. Notably, this association trend exhibits distinct patterns between patients with and without DM. For DM patients, the mortality risk is in a relatively low range when the TyG index is approximately between 8.25 and 9.0; for non-DM patients, the mortality risk is in a relatively low range when the TyG index is approximately between 7.0 and 9.0. Overall, the TyG index exerted a dominant effect on mortality risk in ADHF patients, while CRP further amplified the risk by modulating the effect intensity of TyG index.

### Subgroup analysis stratified by DM and non-DM populations

In ADHF patients with and without DM, we further conducted stratified interaction analyses based on age, gender, LVEF, NYHA classification, and comorbidities (**Table 5**). The results revealed that in non-DM patients, the association between CTI and prognosis exhibited significant interaction effects in gender subgroups, hypertension subgroups, and stroke subgroups. Specifically, the association between CTI levels and all-cause mortality risk was stronger in male patients than in females (*P* for interaction = 0.03). Moreover, the mortality risk was further elevated in subgroups with comorbid hypertension or stroke (All *P* for interaction < 0.05). Notably, among patients with comorbid DM, the prognostic value of CTI demonstrated no subgroup differences, with risk associations remaining consistent across all subgroups (All *P* for interaction > 0.05).

**Table 5:** Stratified analysis showed the relationship between CTI and 30-day mortality in patients with ADHF in different age, gender, NYHA classification, LVEF and whether combined with hypertension/stroke/CHD.

Subgroup	HR (95% CI)	
	Diabetes group	Non-diabetic group
Gender		
Male	4.12 (2.41, 7.06)	3.58 (2.19, 5.87)
Female	2.62 (1.48, 4.64)	1.47 (0.78, 2.76)
*P* for interaction	0.24	0.03
Age (years)		
19-70	2.59 (1.29, 5.17)	2.70 (1.49, 4.88)
71-99	4.24 (2.51, 7.18)	2.82 (1.67, 4.76)
*P* for interaction	0.9587	0.91
NYHA classification		
III	3.02 (1.47, 6.21)	3.37 (1.81, 6.28)
IV	3.48 (2.19, 5.52)	2.25 (1.39, 3.65)
*P* for interaction	0.74	0.30
LVEF		
< 50%	3.72 (1.88, 7.37)	2.33 (1.36, 3.99)
≥ 50%	3.31 (2.02, 5.42)	2.87 (1.66, 4.95)
*P* for interaction	0.77	0.59
Hypertension		
Yes	3.52 (2.20, 5.64)	6.27 (2.74, 14.36)
No	3.01 (1.58, 5.72)	1.96 (1.25, 3.09)
*P* for interaction	0.67	<0.01
Stroke		
Yes	3.48 (1.96, 6.18)	7.01 (2.72, 18.07)
No	3.24 (1.93, 5.44)	2.10 (1.37, 3.24)
*P* for interaction	0.85	0.02
CHD		
Yes	2.80 (1.89, 4.16)	2.40 (1.19, 4.83)
No	3.54 (2.17, 5.77)	2.71 (1.71, 4.30)
*P* for interaction	0.69	0.77

HR, hazard ratios; CI, confidence interval; CTI, C-reactive protein-triglyceride-glucose index; ADHF, acute decompensated heart failure; CHD, coronary heart disease; NYHA, New York Heart Association; LVEF, left ventricular ejection fraction.

Note: Models adjusted for the same covariates as in model III (**Table 2**), except for the stratification variable.

### Comparative performance of the CTI and ADHERE models in mortality prediction

As shown in **Supplementary Table 3**, we compared the predictive performance of the CTI model and the ADHERE model. The study results indicated that the area under the curve of the CTI model was 0.73, which was significantly higher than the 0.64 of the ADHERE model (Delong *P* < 0.05). Continuous net reclassification analysis showed that the CTI model achieved a significant net improvement compared with the ADHERE model, with a net reclassification improvement of 0.18 (*P* < 0.05).

### Sensitivity analysis

The validation results in the cohort of U.S. patients with congestive HF further revealed a positive association between CTI and all-cause mortality, reproducing the core findings of this study (**Supplementary Table 4**). In addition, even after further adjusting for statins, sodium-glucose cotransporter-2 inhibitors, and anti-inflammatory drugs, the main conclusions of this study remained robust (**Supplementary Table 5**). Finally, the analysis based on cardiovascular-specific mortality yielded results consistent with those of all-cause mortality, further supporting the robustness of the study findings (**Supplementary Table 6**).

## Discussion

Based on the Jiangxi-ADHF II cohort study, CTI was confirmed as an independent risk factor for 30-day all-cause mortality in ADHF patients, with its risk assessment value being more pronounced among those with comorbid DM. Notably, heatmaps further demonstrated that the prognostic impact of CTI components exhibited distinct risk patterns between ADHF patients with and without DM.

This study focuses on the complex role of multimorbidity and its clinical translational value, with particular emphasis on the joint lethal effects of HF-DM comorbidity (6–11, 48). The findings demonstrate a significant positive correlation between CTI and short-term mortality risk in ADHF patients, with the risk gradient further amplified in those with comorbid DM. This suggests that metabolic-inflammatory interactions may exacerbate clinical deterioration in comorbid states. This finding aligns closely with the cross-disease risk prediction value of CTI as a systemic biological marker: Xu et al. demonstrated a linear positive correlation between CTI and CHD risk in cardiovascular research (43), while Huo's team and Tang's team independently revealed its positive association with stroke risk across different cohorts (41, 42). In oncology cohorts, Shi et al. reported that elevated CTI conferred a 225% increased 90-day mortality risk in gastrointestinal cancer patients (49), and the INSCOC cancer cachexia cohort further showed that each 1-standard deviation increase in CTI elevated short-term mortality risk by 22% (50). Additional studies have identified significant associations between CTI and multisystem endocrine-metabolic/immune regulatory disorders (51–55), collectively suggesting CTI's strong generalizability as a trans-disease risk stratification tool.

This study employed Cox regression models, combined with RCS, and heatmaps, to systematically elucidate the joint effects of the CTI and its components (TyG index and CRP) on all-cause mortality risk in ADHF patients with DM comorbidity. Among ADHF patients with comorbid DM, CTI demonstrated a significant nonlinear positive association with 30-day all-cause mortality risk (*P* for non-linearity = 0.02), manifesting as a threshold effect; whereas in non-DM ADHF patients, a linearly increasing risk pattern was observed (*P* for non-linearity = 0.72). The heatmap results further elucidated: We found that the lowest mortality risk occurred when the TyG index was within a certain range concurrently with a CRP level under 50 mg/L, a pattern consistent in both DM and non-DM patients. This finding is closely aligned with the theoretical threshold of metabolic homeostasis in chronic diseases (56–64). Notably, the longitudinal CRP gradient effect demonstrated a linear association, with all-cause mortality risk in ADHF patients progressively increasing alongside rising CRP levels. Furthermore, when comparing ADHF patients with DM to those without DM, the former exhibited relatively higher CRP levels under equivalent mortality risk. This discrepancy may be attributed to the DM microenvironment, which potentially amplifies inflammatory signaling transduction efficiency through increased accumulation of advanced glycation end-products (65). At the level of interaction between the TyG index and CRP, joint effect analysis revealed that the TyG index exerted a dominant effect on mortality risk, while CRP further amplified this risk by modulating the magnitude of the TyG index's effect. Specifically, when the TyG index exceeded 9 and CRP levels progressively increased, patients exhibited a significantly elevated 30-day mortality risk compared to baseline levels. This joint effect may stem from the vicious cycle formed between TyG index-induced IR and CRP-mediated chronic inflammation. This cycle escalates mortality risk through multiple pathways, including disrupting myocardial energy metabolism and exacerbating mitochondrial dysfunction (12–15).

This study further revealed population heterogeneity in the association between CTI and 30-day all-cause mortality risk in ADHF patients through subgroup analysis. Specifically, among ADHF patients without DM, CTI-related mortality risk demonstrated gender- and comorbidity-specific stratification characteristics: male patients exhibited significantly higher mortality risk compared to females, and this risk was further elevated in patients with comorbid hypertension or stroke. Previous studies have consistently validated the survival advantage of female HF patients, with this gender-based benefit persisting across different ejection fraction subtypes (66, 67). The underlying mechanisms can be elucidated from multiple dimensions: Biologically, analyses of sample data revealed that male patients exhibit significantly higher median CTI values compared to females, which may be attributed to male-specific visceral fat accumulation patterns and the cardioprotective effects of estrogen in females (68, 69). Behaviorally, adverse lifestyle factors such as smoking and drinking consumption habits are more prevalent in males compared to females, which further exacerbates the risk of future adverse events (70–72). In terms of comorbidity modification effects, patients with comorbid hypertension or stroke exhibit higher CTI levels. This phenomenon might be driven by enhanced IR and systemic inflammation associated with comorbid conditions (73–76). Additionally, the comorbid states of hypertension or stroke inherently contribute to further elevated mortality risk (77–79).

The value of this study lies in providing a novel and actionable strategy for the clinical management of ADHF. Owing to its simplicity and cost-effectiveness, the CTI possesses significant advantages for clinical translation (40–43). We advocate for its systematic integration at the frontline of patient management to establish an evidence-based risk stratification system. The core applications include: embedding an automated CTI assessment module in the information system to achieve early warning and precise intervention for high-risk ADHF patients; meanwhile, integrating it with mature risk models or Artificial Intelligence-assisted systems to enable dynamic, intelligent mortality risk prediction, thereby paving the way for smarter clinical pathways.

### Strengths and limitations

This study represents a positive association between CTI and all-cause mortality risk in ADHF patients, and further reveals that DM comorbidity significantly amplifies this correlation. Additionally, through the heatmap visualization analysis, we have further investigated the relationship between CTI components (TyG index and CRP) and mortality risk. These findings on cross-domain biomarkers offer a new framework for personalizing risk management in ADHF, informing clinical decision-making.

The study has several limitations: (1) As a regional observational cohort study, the generalizability of findings may be limited by regional and ethnic constraints, necessitating validation in large-scale multicenter cohorts before broader extrapolation; (2) Although multiple confounders have been corrected for, unmeasured residual confounders may still affect the effect estimates; (3) The dynamic impacts of metabolic-modulating medications on TyG index and CRP levels were not quantitatively assessed, which may result in systematic underestimation of the true exposure-outcome associations. (4) Since CRP is not a routine test for HF patients, a large number of cases with missing CRP data were consequently excluded from our analysis. This may have introduced selection bias and could affect the generalizability of the results. This limitation requires further validation in future studies. (5) While 30-day follow-up effectively captures acute-phase events, it may underestimate the time-dependent associations between metabolic dysfunction, inflammation, and long-term prognosis. Future studies should employ extended follow-up duration with dynamic predictive modeling to elucidate longitudinal effect trajectories, and incorporate additional study outcomes (such as readmission rates) to enhance the applicability of the findings.

## Conclusion

This study supports the clinical value of CTI as a novel biomarker for short-term mortality in ADHF patients and elucidates the enhancing effect of DM comorbidity on this positive association. Further heatmap analyses suggest that controlling TyG and CRP levels in ADHF patients, particularly those with DM comorbidity, may significantly reduce short-term all-cause mortality. These findings underscore the pivotal role of the metabolic-inflammatory pathway in ADHF progression, offering valuable references for early risk stratification and targeted therapeutic interventions in ADHF management.

## Data Availability

The raw data supporting the conclusions of this article will be made available by the authors, without undue reservation.
