# Supplementary material for: Inflammatory storm and metabolic disorders: unraveling heterogeneity in mortality risk for comorbid diabetes mellitus and heart failure via the C-reactive protein-triglyceride-glucose index
Source: Front Endocrinol (Lausanne). 2025 Nov 19;16:1689238. doi: 10.3389/fendo.2025.1689238 (PMC12672285; doi:10.3389/fendo.2025.1689238)
Supplement: Supplementary file 1 [file DataSheet1.docx]

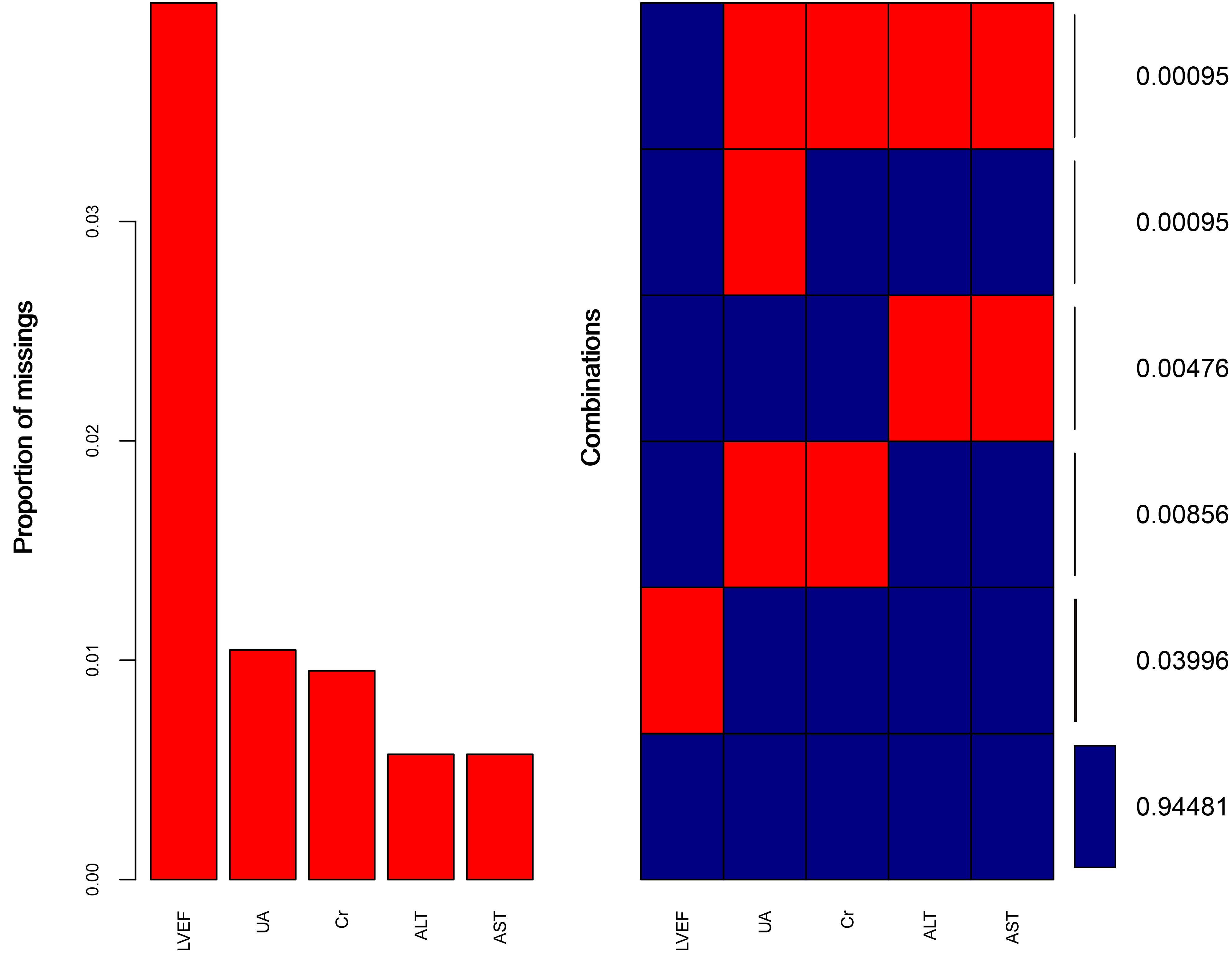


**Supplementary Figure 1:** Missing data cross-information diagram. LVEF: left ventricular ejection fraction; Cr: creatinine; ALT: alanine aminotransferase; AST: aspartate aminotransferase; UA: uric acid.
