# Supplementary material for: Inflammatory storm and metabolic disorders: unraveling heterogeneity in mortality risk for comorbid diabetes mellitus and heart failure via the C-reactive protein-triglyceride-glucose index
Source: Front Endocrinol (Lausanne). 2025 Nov 19;16:1689238. doi: 10.3389/fendo.2025.1689238 (PMC12672285; doi:10.3389/fendo.2025.1689238)
Supplement: Supplementary file 2 [file Table1.docx]

Supplementary Table 1. The missing number and rate of covariates.

|  | Non- Missing | Missing |
| --- | --- | --- |
| Gender | 1051 | 0 |
| Age | 1051 | 0 |
| Hypertension | 1051 | 0 |
| DM | 1051 | 0 |
| Stroke | 1051 | 0 |
| CHD | 1051 | 0 |
| NYHA classification | 1051 | 0 |
| Drinking status | 1051 | 0 |
| Smoking status | 1051 | 0 |
| LVEF | 1009 | 42 |
| ALT | 1045 | 6 |
| AST | 1045 | 6 |
| Cr | 1041 | 10 |
| UA | 1040 | 11 |
| TG | 1051 | 0 |
| TC | 1051 | 0 |
| HDL-C | 1051 | 0 |
| LDL-C | 1051 | 0 |
| FPG | 1051 | 0 |
| NT-proBNP | 1051 | 0 |
| TyG index | 1051 | 0 |
| CRP | 1051 | 0 |
| CTI | 1051 | 0 |

Abbreviations as in Table 1.

Supplementary Table 2: Collinearity diagnostics steps.

|  | VIF | | |
| --- | --- | --- | --- |
|  | Step 1 | Step 2 | Step 3 |
| CTI | 5.9 | 4.5 | 4.5 |
| Gender | 1.2 | 1.2 | 1.2 |
| Age | 1.3 | 1.3 | 1.3 |
| Hypertension | 1.2 | 1.2 | 1.2 |
| Stroke | 1.1 | 1.1 | 1.1 |
| CHD | 1.1 | 1.1 | 1.1 |
| NYHA classification | 1.1 | 1.1 | 1.1 |
| Drinking status | 1.5 | 1.5 | 1.4 |
| Smoking status | 1.5 | 1.5 | 1.5 |
| LVEF | 1.2 | 1.2 | 1.2 |
| ALT | **6.1** | **6.1** | **NA** |
| AST | 6.1 | 6.1 | 1.1 |
| Cr | 1.4 | 1.4 | 1.4 |
| UA | 1.3 | 1.3 | 1.3 |
| TG | 5.7 | 2.4 | 2.4 |
| TC | 1.9 | 1.9 | 1.9 |
| HDL-C | 1.8 | 1.7 | 1.7 |
| LDL-C | 1 | 1 | 1 |
| FPG | 3.4 | 1.6 | 1.6 |
| NT-proBNP | 1.3 | 1.2 | 1.2 |
| TyG index | **12.2** | **NA** | **NA** |
| CRP | 2.7 | 2.3 | 2.3 |

VIF: variance inflation factor; VIF = 1/(1-R^2^). Abbreviations as in Table ​1.

Note: The variables with VIF>5 will be regarded as collinear variables.

Supplementary Table 3: Predictive Performance of the CTI versus ADHERE model.

|  | ADHERE model | CTI Model | improve | *P*-value |
| --- | --- | --- | --- | --- |
| AUC | 0.64 | 0.73 | 0.09 | 0.03 |
| continuous-NRI | - | - | 0.18 (0.03, 0.32) | 0.02 |

AUC: area under the curve; NRI: net reclassification improvement; other abbreviations as in Table ​1.

Supplementary Table 4: Multivariable Cox regression analysis of the association between CTI and mortality in a US cohort with congestive heart failure.

|  | HR (95% CI) |
| --- | --- |
| CTI | 2.43 (1.88, 3.14) |
| CTI tertiles |  |
| T1(Low) | 1.0 |
| T2(Moderate) | 0.91 (0.44, 1.90) |
| T3(High) | 2.35 (1.22, 4.56) |

Abbreviations: HR: hazard ratios; CI: confidence interval; ADHF: acute decompensated heart failure; CTI: C-reactive protein-triglyceride-glucose index.

Adjusted for gender, age, drinking status, smoking status, hypertension, and CHD, RBC, PLT, AST, Cr, UA, LDL-C.

Supplementary Table 5: Multivariable Cox regression analysis of the associations between CTI and 30-day mortality in patients with ADHF.

|  | HR (95% CI) |
| --- | --- |
| CTI | 2.43 (1.88, 3.14) |
| CTI tertiles |  |
| T1(Low) | 1.0 |
| T2(Moderate) | 0.91 (0.44, 1.90) |
| T3(High) | 2.35 (1.22, 4.56) |

Abbreviations: HR: hazard ratios; CI: confidence interval; ADHF: acute decompensated heart failure; CTI: C-reactive protein-triglyceride-glucose index.

Adjusted for gender, age, drinking status, smoking status, hypertension, stroke and CHD, NYHA classification, LVEF, RBC, PLT, AST, Cr, UA, LDL-C, NT-proBNP, Statin therapy, SGLT-2 therapy and Anti-inflammatory therapy.

Supplementary Table 6: Multivariable Cox regression analysis of the associations between CTI and 30-day cardiovascular mortality in patients with ADHF.

|  | HR (95% CI) |
| --- | --- |
| CTI | 2.35 (1.73, 3.18) |
| CTI tertiles |  |
| T1(Low) | 1.0 |
| T2(Moderate) | 1.12 (0.51, 2.44) |
| T3(High) | 2.47 (1.19, 5.14) |

Abbreviations: HR: hazard ratios; CI: confidence interval; ADHF: acute decompensated heart failure; CTI: C-reactive protein-triglyceride-glucose index.

Adjusted for gender, age, drinking status, smoking status, hypertension, stroke and CHD, NYHA classification, LVEF, RBC, PLT, AST, Cr, UA, LDL-C and NT-proBNP.
